# Tannase-Converted Green Tea Extract with High (−)-Epicatechin Inhibits Skeletal Muscle Mass in Aged Mice

**DOI:** 10.1155/2020/4319398

**Published:** 2020-01-29

**Authors:** Ki-Bae Hong, Hee-Seok Lee, Dong Hyeon Kim, Joo Myung Moon, Yooheon Park

**Affiliations:** ^1^BK21 Plus, College of Health Science, Korea University, Seoul 02841, Republic of Korea; ^2^Department of Food Science and Technology, Chung-Ang University, Anseong 17546, Republic of Korea; ^3^BTC Corporation, Technology Development Center, No. 703.705 Haean-ro Sangnok-gu Ansan-si, Gyeonggi-do, Republic of Korea; ^4^Department of Food Science and Biotechnology, Dongguk University, Goyang 10326, Republic of Korea

## Abstract

The objective of this study was to investigate the effects of tannase-converted green tea extract on body composition, muscle oxidative stress-related factors, and differentiation-related factors. The mean bone-related parameters and body composition were determined by the live dual-energy X-ray absorptiometry (DEXA). Quantitative real-time polymerase chain reaction (qRT-PCR) and western blotting were used to determine mRNA expression and protein levels, respectively. The results of total mass testing in the epicatechin control (EC) and middle concentration tannase-converted green tea extract (*T*_1_) intake groups were not significantly different compared with those in the control group; however, the high-concentration tannase-converted green tea extract (*T*_2_) group showed a significantly higher effect to the lean than that of all other groups (*p* < 0.05). The results of the assay of muscle differentiation-related genes indicated that the expression levels in the EC and *T*_1_ groups (*p* < 0.05) and the expression levels in the *T*_2_ group (*p* < 0.01) were significantly different in the bicep femoris compared with that in the control group. The results of the SOD gene assay indicate that the expression levels in the EC and *T*_1_ groups (*p* < 0.05) and the expression level in the *T*_2_ group (*p* < 0.01) were significantly different in the bicep femoris compared with that in the control group. Additionally, SOD gene expression in the *T*_2_ group was significantly increased (*p* < 0.05) in the soleus compared with that in the control, EC and *T*_1_ groups. Our results suggest that tannase-converted green tea extract prevents muscle loss and regulates the quantity and quality of muscle by the levels of antioxidant stress-related enzymes and muscle differentiation factors to a greater extent than the administration of epicatechin and middle dose green tea extract.

## 1. Introduction

*Camellia sinensis*, a traditional East Asian tea material, has been extensively used for the past few decades and has been officially recognized in most countries. Green tea has been adequately studied to meet the standards required to support its use globally. Green tea contains a number of polyphenolic compounds known as catechins with various physiological activities, such as antioxidant, antihyperglycemic, immunostimulatory, and antitumor effects [1, 2]. Polyphenolic compounds and polysaccharides known to stimulate various physiological activities and green tea extract have been considered a potential new source of bioactive agents [3].

Plant cell walls have important effects on the physical properties of fruits and vegetables and are complex in which the polysaccharides, phenolic components, and proteins are linked together by covalent and noncovalent bonds [4, 5]. Enzyme treatment may induce degradation and structural modifications that lead to changes in polysaccharides, phenolic compounds, and proteins. As a result, enzyme-treated material is dehydrated, has an increased volume, undergoes changes in texture, and has altered functional components [6, 7]. Currently, changes in green tea induced by treatment with various enzymes and the enzyme treatment are essential to introduce a new extraction process to increase the value of green tea [8, 9].

Sarcopenia is the result of the production of reactive oxygen species (ROS) that can interfere with homeostasis and cause muscle loss in the antioxidant defense system of the body [10, 11]. Studies addressing the inhibition of muscles loss are important not only for athletes but also for less physically active older people and active young people. Pharmacological drugs or treatments have not been effective for treating muscle loss. In recent years, there has been a growing interest in using natural supplements to reduce physical fatigue and muscle loss. Moreover, various methods have been used to enhance the antioxidant activity of natural substances and to reduce muscle loss due to aging. Several strategies, including enzymatic conversion to increase the amount of polyphenols or enzyme-treated extraction to enhance polyphenol extraction, have been developed [12].

For instance, expansion of green tea may increase the extraction levels of polyphenols and flavonoids, leading to the significant changes in the antioxidant content. However, studies showing the effects of the extract of enzyme-treated tea on sarcopenia are very limited. This study was designed to identify whether tannase-converted green tea extract enhances muscle health via regulation of mRNA expression and protein levels. We used live dual-energy X-ray absorptiometry (DEXA), quantitative real-time polymerase chain reaction (qRT-PCR), and western blotting to investigate the effects of tannase-converted green tea extract in aged mice.

## 2. Materials and Methods

### 2.1. Samples

**Figure 1:**
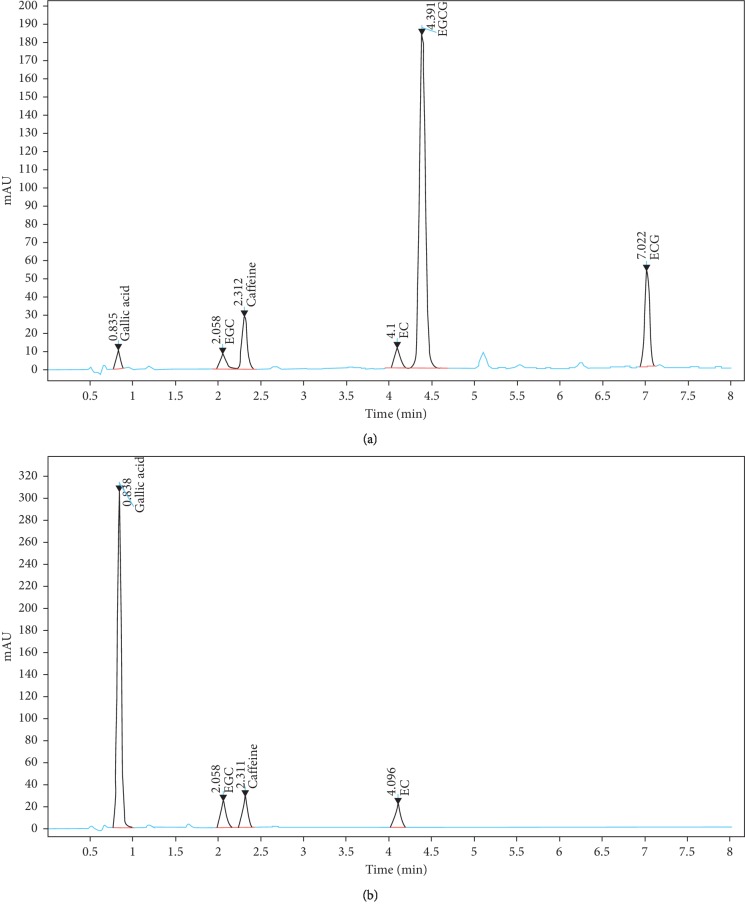
High-performance liquid chromatography chromatogram for catechins in green tea extract (a) and tannase-converted green tea extract under optimum extraction condition (b). GC, (–)-gallocatechin; EGC, (–)-epigallocatechin; EGCG, (–)-epigallocatechingallate; EC, (–)-epicatechin; GCG, (–)-gallocatechingallate; ECG, (–)-epicatechingallate.

Tannase-converted green tea extract was obtained from BTC Co. Ltd. (Ansan, South Korea). The HPLC analytical grade standard of epicatechin (EC) was purchased from Sigma-Aldrich (St. Louis, MO, USA). The clear supernatant obtained from the green tea extract was used as a substrate for enzymatic conversion by tannase (Kikkoman Biochemifa, Tokyo, Japan). The characteristics of the green tea extract and tannase-converted green tea extract are shown in Figure 1.

### 2.2. Animals

Male ICR mice (24-month-old) were purchased from Orient Bio (Orient Bio Inc., Seongnam, Korea). All animals were caged at 22 ± 2°C and 55 ± 5% humidity with a 12 h light/dark cycle. Normal pellet diet and water were provided ad libitum. In this study, all animal experimental protocols were approved by the Dongguk University Animal Care Committee. The experimental groups consisted of the following: (1) control diet (AIN 93M, *n* = 8), (2) epicatechin diet (EC, AIN 93M + epicatechin 2 mg/kg, *n* = 8), (3) middle concentration tannase-converted green tea extract diet (*T*_1,_ AIN 93M + tannase-converted green tea extract 20 mg/kg, *n* = 8), and (4) high concentration tannase-converted green tea extract (*T*_2,_ AIN 93M + tannase-converted green tea extract 40 mg/kg, *n* = 8).

### 2.3. Measurements of Body Composition

The mean bone-related parameters and body composition were determined by live dual-energy X-ray absorptiometry (DEXA; InAlyzer, Medikors, Seungnam, Republic of Korea). Animals were made to fast overnight and were injected intraperitoneally with ketamine (100 mg/kg) and xylazine (10 mg/kg) for anesthesia. Bone mass and bone mineral content were expressed as the amounts of the bone content and the bone mineral content. Bone mineral density was expressed as total average of the bone mineral density. In addition, bone area and volume were determined by using total surface area and volume of the bone. Fat mass and lean mass were expressed as total amount of the fat and lean contents, and tissue percentage fat was calculated as the ratio of fat content to total body mass.

### 2.4. mRNA Extraction and qRT-PCR Analysis

The TRIzol® reagent (Invitrogen, Carlsbad, CA, USA) was used for total RNA isolation, according to the manufacturer's protocol, and qRT-PCR analysis was performed on biceps femoris and soleus. One microgram of total RNA was treated with RQ1 RNase-free DNase I (Promega, WI, USA) and reverse transcribed using SuperScript® III reverse transcriptase (Invitrogen), using oligo (dT) primer. Real-time PCR (qRT-PCR) was performed using the Taqman gene expression master mix (Applied Biosystems, CA, USA), and quantitative analyses were conducted using the StepOne plus Software V. 2.0 (Applied Biosystems). All the results were determined based on a validated control gene, GAPDH (NM_001289726.1), using the ΔΔCt method. Information for target genes used in qRT-PCR is as follows: MyoD (NM_010866.2), Myf5 (NM_008656.5), Myogenin (NM_031189.2), FOXO1 (NM_019739.3), FOXO3 (NM_019740.2), SOD (NM_011434.1), CAT (NM_009804.2), and GST (NM_001251762.2).

### 2.5. Western Blotting

The bicep femoris and soleus were lysed for 30 min and centrifuged at 12,000 ×g for 10 min at 4°C. The protein concentration was quantified using BSA (bovine serum albumin) as the standard. The lysate (10 *μ*g) was separated using 10% Mini-protean TGXTM gels and transferred to a polyvinylidene difluoride (PVDF) membrane at 100 V for 1 h. The membrane was blocked with a TBST (0.1% Tween 20 + TBS) solution containing 5% skim milk for 1 h. The primary antibody was diluted with skim milk (1 : 1000), and the reaction was incubated overnight at 4°C; the membrane was washed 3 times using TBST. Protein expression was detected with a specific antibody using a ChemiDoc™ imaging system (Bio-Rad, Hercules, CA), and the results were normalized to a validated control gene, *β*-actin. Primary antibodies were purchased from Abcam (Cambridge, UK), Santa Cruze Biotechnology (Santa Cruz, CA, USA), and Cell Signaling Technology (Danvers, MA, USA), and the target protein used for western blotting were as follows: p70 S6 kinase (pS6K), the mammalian target of rapamycin (mTOR), follistatin, forkhead box protein O3a (FOXO3a), myostatin, muscle RING finger protein-1 (MuRF-1), and atrogin-1.

### 2.6. Statistical Analysis

All analyses were performed using the R-software (version 3.2.5, the *R* Foundation, Vienna, Austria). The *p* values were derived from Tukey's multiple-range test, and values of *p* < 0.05 were considered to be statistically significant. Values are expressed as the mean ± standard error (SE) for each group, and all experiments were repeated 4 times.

## 3. Results

### 3.1. Effects of Tannase-Converted Green Tea Extract on Body Composition

**Figure 2:**
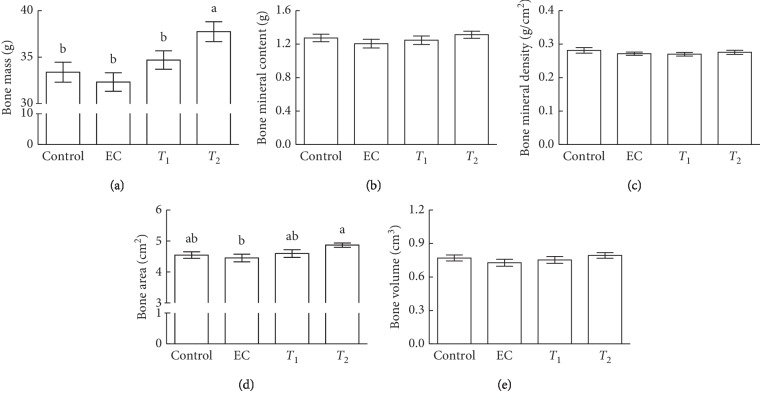
Effects of tannase-treated catechin on (a) bone mass, (b) bone mineral content, (c) bone mineral density, (d) bone area, and (e) bone volume in aged mice. Each value represents the mean ± standard error (SE) for each group (*n* = 8). Different letters indicate significant differences at *p* < 0.05  according to Tukey's test. EC: epicatechin (2 mg/kg); *T*_1_: middle concentration tannase-converted green tea extract (20 mg/kg); *T*_2_: high concentration tannase-converted green tea extract (40 mg/kg).

**Figure 3:**
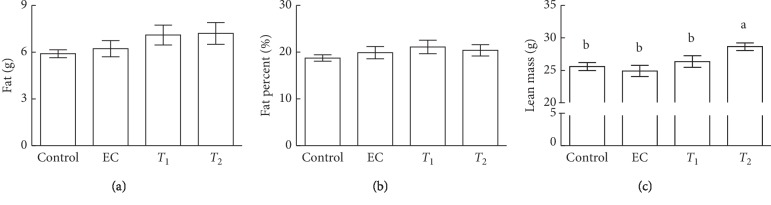
Effects of tannase-treated catechin on (a) fat, (b) fat percent, and (c) lean mass in aged mice. Each value represents the mean ± standard error (SE) for each group (*n* = 8). Different letters indicate significant differences at *p* < 0.05  according to Tukey's test. EC: epicatechin (2 mg/kg); *T*_1_: middle concentration tannase-converted green tea extract (20 mg/kg); *T*_2_: high-concentration tannase-converted green tea extract (40 mg/kg).

The effects of tannase-converted green tea extract were investigated by measuring the body composition of aged mice (Figures 2 and 3). Results from DEXA analysis showed that *T*_2_ mice exhibited significantly increased bone mass compared with control, EC, and *T*_1_ mice (Figure 2(a), *p* < 0.05). *T*_2_ mice had significantly increased bone area compared with EC mice (Figure 2(d), *p* < 0.05). Additionally, *T*_2_ treatment showed significantly increased lean mass when compared with control, EC, and *T*_1_ groups (Figure 3(c), *p* < 0.05). The intake of EC, *T*_1_, and *T*_2_ did not induce any significant differences in bone mineral content, bone mineral density, the bone volume, fat mass, and fat percent compared with those in the control group.

### 3.2. Effects of Tannase-Converted Green Tea Extract on the Transcription Levels of Muscle Differentiation-Related Factors

**Figure 4:**
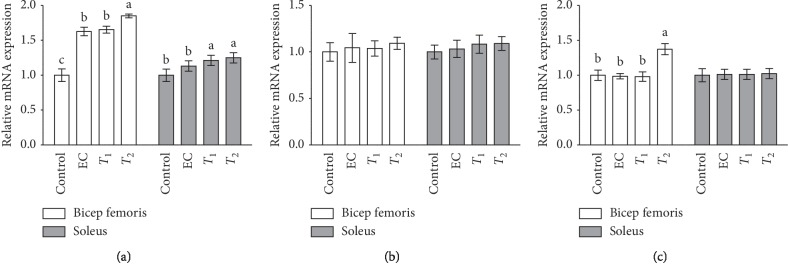
Effects of tannase-treated catechin on mRNA levels of (a) MyoD, (b) Myf5, and (c) myogenin in aged mice. Each value represents the mean ± standard error (SE) for each group (*n* = 8). Different letters indicate significant differences at *p* < 0.05 according to Tukey's test. EC: epicatechin (2 mg/kg); *T*_1_: middle-concentration tannase-converted green tea extract (20 mg/kg); *T*_2_: high-concentration tannase-converted green tea extract (40 mg/kg); MyoD: myoblast determination protein; Myf5: myogenic factor 5.

The effects of tannase-converted green tea extract on the transcription levels of muscle differentiation-related factors are presented in Figures 3 and 4. The MyoD level in the EC and *T*_1_ groups (*p* < 0.05) and the expression levels in the *T*_2_ group (*p* < 0.01) were significantly different in the bicep femoris compared with that in the control group. In the soleus, the gene expression levels in the *T*_1_ and *T*_2_ groups were significantly increased (*p* < 0.05) compared with those in the control and EC groups. In the case of the Myf5 gene, there were no significant differences in its expression levels between the EC, *T*_1_, and *T*_2_ groups in the bicep femoris and soleus compared with that in the control group. In the case of myogenin gene, the expression levels of EC and *T*_2_ groups were significantly increased (*p* < 0.05) in the bicep femoris compared with those in the control and *T*_1_ groups. In the case of the FOXO1 gene, the expression levels in the *T*_2_ group were significantly reduced in the bicep femoris compared with that in the control group, EC, and *T*_1_ groups. The expression levels of the FOXO1 gene in the soleus and the expression of the FOXO3 gene in the bicep femoris and soleus did not show any significant differences between the groups.

### 3.3. Effects of Tannase-Converted Green Tea Extract on the Transcription Levels of Muscle Oxidative Stress-Related Factors

**Figure 5:**
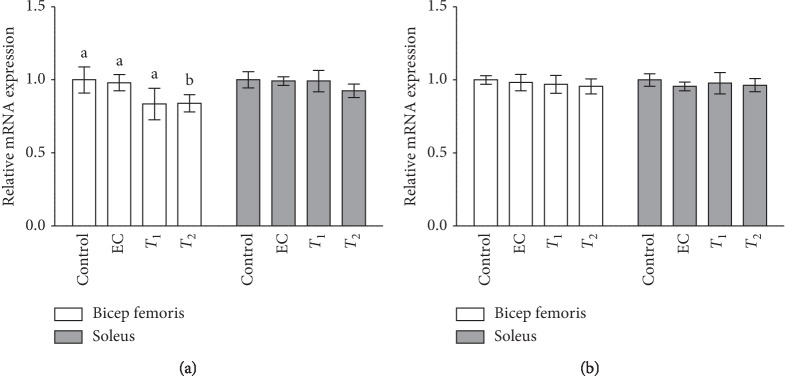
Effects of tannase-treated catechin on mRNA levels of (a) FOXO1 and (b) FOXO3 in aged mice. Each value represents the mean ± standard error (SE) for each group (*n* = 8). Different letters indicate significant differences at *p* < 0.05 according to Tukey's test. EC: epicatechin (2 mg/kg); *T*_1_: middle-concentration tannase-converted green tea extract (20 mg/kg); *T*_2_: high-concentration tannase-converted green tea extract (40 mg/kg); FOXO1-3: forkhead box protein O1-3.

**Figure 6:**
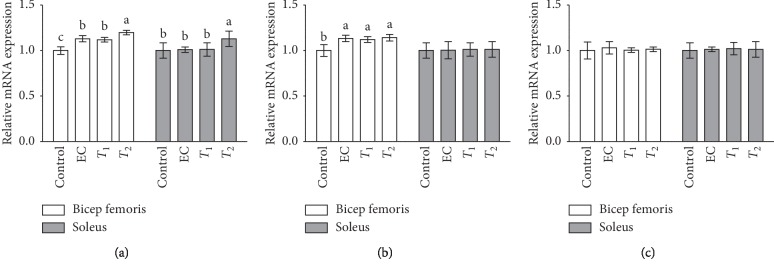
Effects of tannase-treated catechin on mRNA levels of (a) SOD, (b) CAT, and (c) GST in aged mice. Each value represents the mean ± standard error (SE) for each group (*n* = 8). Different letters indicate significant differences at*p* < 0.05 according to Tukey's test. EC: epicatechin (2 mg/kg); *T*_1_: middle concentration tannase-converted green tea extract (20 mg/kg); *T*_2_: high-concentration tannase-converted green tea extract (40 mg/kg); SOD: superoxide dismutase; CAT: catalase; GST: glutathione S-transferase.

The effects of tannase-converted green tea extract on oxidative stress-related mRNA levels in aged mice are shown in Figures 5 and 6. In the case of SOD gene, the expression levels in the EC and *T*_1_ groups (*p* < 0.05) and the expression levels in the *T*_2_ group (*p* < 0.01) were significantly different in the bicep femoris with those in the control group. Additionally, the SOD gene expression levels in the *T*_2_ group were significantly increased in the soleus (*p* < 0.05) compared with that in the control, EC, and *T*_1_ groups. In the case of the CAT gene, the expression levels in the EC, *T*_1_, and *T*_2_ groups were significantly increased in the bicep femoris compared with those in the control group. The expression levels of the CAT gene in soleus and the GST gene in the bicep femoris and soleus did not show any significant differences between the groups.

### 3.4. Effects of Tannase-Converted Green Tea Extract on Protein Levels of Muscle Differentiation Factors

**Figure 7:**
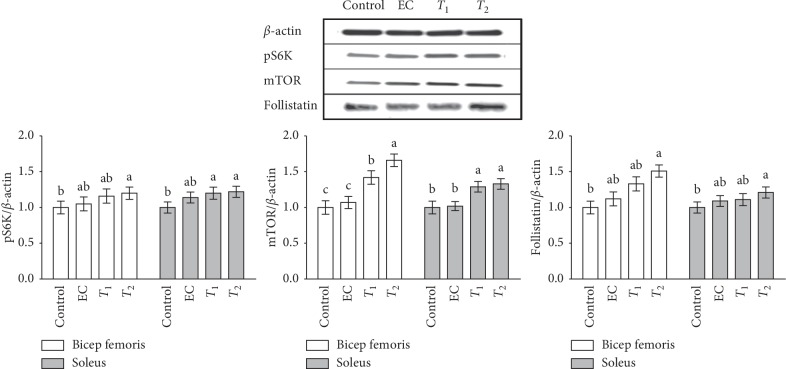
Effects of tannase-treated catechin on the protein expression of pS6K, mTOR, and follistatin in aged mice. Each value represents the mean ± standard error (SE) for each group (*n* = 8). Different letters indicate significant differences at *p* < 0.05 according to Tukey's test. EC: epicatechin (2 mg/kg); *T*_1_: middle concentration tannase-converted green tea extract (20 mg/kg); *T*_2_: high-concentration tannase-converted green tea extract (40 mg/kg); pS6K: p70 S6 kinase; mTOR: the mammalian target of rapamycin.

**Figure 8:**
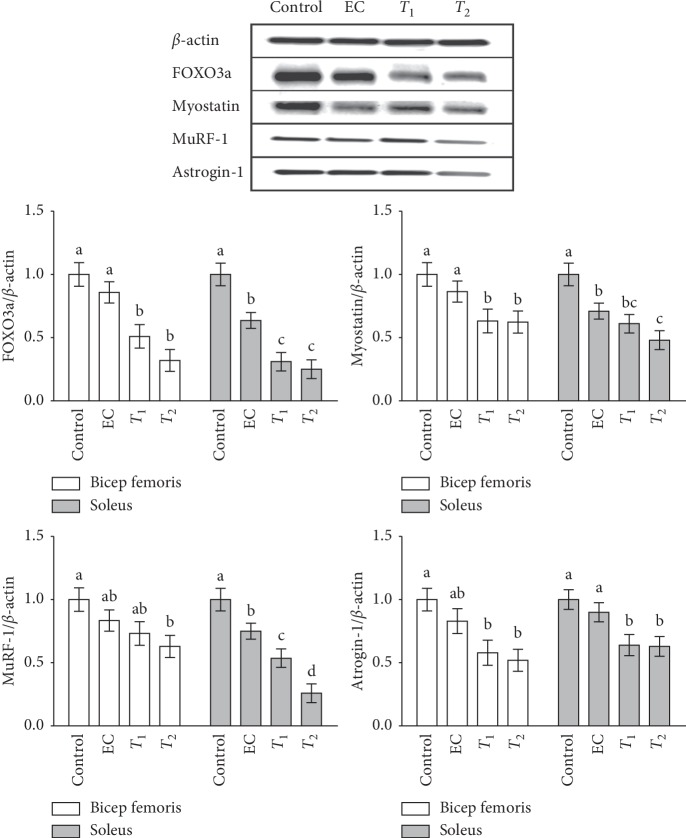
Effects of tannase-treated catechin on the protein expression of FOXO3a, myostatin, MuRF-1, and atrogin-1 in aged mice. Each value represents the mean ± standard error (SE) for each group (*n* = 8). Different letters indicate significant differences at *p* < 0.05 according to Tukey's test. EC: epicatechin (2 mg/kg); *T*_1_: middle-concentration tannase-converted green tea extract (20 mg/kg); *T*_2_: high-concentration tannase-converted green tea extract (40 mg/kg); FOXO3a: forkhead box protein O3a; MuRF-1: muscle RING finger protein-1.

To investigate whether the mechanism of the tannase-converted green tea extract involves multiple protein processes, the levels of proteins were determined (Figures 7 and 8) In the case of pS6K, there were no significant differences in the bicep femoris between the control, EC, and *T*_1_ groups; however, protein expression in the *T*_2_ group was significantly increased compared with that in the control group. In the soleus, there was a significant difference in the expression levels in the *T*_1_ and *T*_2_ groups compared with that in the control group (*p* < 0.05). In the case of mTOR, a significant increase in protein expression was observed in the bicep femoris in the EC and *T*_1_ groups (*p* < 0.05) and *T*_2_ group (*p* < 0.01) compared with that in the control group. In the soleus, the protein expression in medium and high concentration of tannase-converted green tea extract was also significantly increased (*p* < 0.05). In the case of follistatin, the EC and *T*_1_ groups did not have any significant difference in the bicep femoris and soleus; however, protein expression in the *T*_2_ group was significantly increased (*p* < 0.01). In case of FOXO3a, there was a significant decrease in the target protein (*p* < 0.05) in the medium and high concentration tannase-converted green tea extract groups (*p* < 0.05) in the bicep femoris compared with that the control and EC groups. In the soleus, the protein expression levels in the EC and *T*_1_ groups (*p* < 0.05) and in the *T*_2_ group (*p* < 0.01) were significantly different. In case of myostatin, significantly decreased protein expression in the bicep femoris was observed in the *T*_1_ and *T*_2_ groups (*p* < 0.05) compared with that in the control and EC groups. In the soleus, the protein expressions levels were significantly different between the EC, *T*_1_, and *T*_2_ groups. In the case of MuRF-1, there was a significant difference (*p* < 0.05) in the bicep femoris and the control group in the *T*_2_ group (*p* < 0.05). The protein expression levels were significantly different in the *T*_1_ group (*p* < 0.01) and the *T*_2_ group (*p* < 0.01). In the case of atrogin-1, there was a significant decrease in the protein levels (*p* < 0.05) in the bicep femoris compared with that in the control group (*p* < 0.05). In the case of the soleus, the levels in the *T*_1_ and *T*_2_ groups were significantly decreased (*p* < 0.05).

## 4. Discussion

Enzymatic hydrolysis is a commonly used processing technique for extracting active compounds from food materials. After enzyme treatment, the yield and total sugar are increased, and this process may promote internal macromolecular breakage, resulting in the simultaneous release of polyphenols, flavonoids, and other chemicals. Tannase (tannin acyl hydrolase, EC 3.1.1.20) is known to catalyze the hydrolysis of the ester and depside bonds in hydrolysable tannins or gallate esters, such as EGCG or ECG in green tea [13]. EGCG is hydrolyzed into EGC and gallic acid (GA) by tannase, and ECG is hydrolyzed into EC and GA [14]. Green tea has been known for various health-promoting benefits, including immunomodulatory, antioxidant, and antifatigue effects [15–17]. Moreover, the bioactive compounds of the green tea extract treated by commercial enzymes reduce various disease risks and are involved in various physiological activities such as anticancer, antiinflammatory, and antioxidant functions [18, 19]. The results of human trials suggest that the chemopreventive effects of green tea depend on (1) its antioxidant action, (2) its specific induction of detoxifying enzymes, and (3) its molecular regulatory functions in cellular growth, development, and apoptosis [20]. Various effects of green tea extract have been reported in invertebrates, vertebrates, and cell lines [1, 21–24].

In the present study, we demonstrated the effects of tannase-converted green tea extract on the mean bone-related parameters and body composition using a DEXA scan (Figures 2 and 3). In estrogen-deficient rats, catechin-rich oil palm leaf extract increased the bone density and structure in a dose-dependent manner, suggesting that catechins can be used as a potential inexpensive ingredient in osteoporosis and bone metabolism [25]. Moreover, daily consumption of a tea containing 690 mg of catechins resulted in a significant reduction in weight, body mass index, and fat mass and subcutaneous fat [26]. To identify the changes in the expression of the muscle differentiation-related genes induced by tannase-converted green tea extract administration, qRT-PCR was carried out and the results of the analysis are shown in Figure 4. Kim et al. substantiated that catechins, such as ECG and EGCG, stimulate muscle regeneration by inducing the Myf5 transcription factor in the satellite cells [27]. Additionally, supplementation with EC appears to act as a muscle growth agent through modulation of myogenic conversion and differentiation-related promyogenic signaling factors [28].

An increase in the levels of ROS and inflammation in skeletal muscle is a result of muscle contraction; high levels of ROS promote contractile dysfunction, leading to age-related muscle atrophy [29, 30]. Antioxidants in green tea extract may decrease the exercise-induced ROS production and improve fatigue resistance [31]. In this study, we confirmed that the transcription factor levels and antioxidant enzyme activities are enhanced by the enzyme-treated green tea extract (Figures 5 and 6). Numerous studies have reported that aging promotes the generation of free radicals, such as ROS, which cause fatigue and reduce the performance of the body, and antioxidant enzymes are used as indicators of the free radical-scavenging capacity [32]. SOD, CAT, and GST are the main enzymes of the antioxidant defense system that eliminates ROS and alleviates muscle loss due to aging [29]. These enzymes inhibit muscle loss by eliminating free radicals, especially ROS, and reducing the production of MDA, thereby protecting the cell structure and reducing muscle loss. Latres et al. showed that phosphorylated FOXO transcription factors inhibit the expression of atrophy-related genes such as atrogin-1 and MuRF-1 [33].

Tannase treatment of green tea extract can enhance muscle endurance by promoting the expression of the key regulators in the skeletal muscle. pS6K, mTOR, follistatin, FOXO3a, myostatin, MuRF-1, and atrogine-1 can induce considerable changes in the skeletal muscle metabolism. We used western blot analysis to determine the biological effects of tannase-converted green tea extract administration on the expression of a number of proteins (Figures 7 and 8). Myostatin is a member of the transforming growth factor-*β* protein group that binds to the activin-IIb receptor, which is secreted and expressed in the skeletal muscle and inhibits skeletal muscle growth [34]. Follistatin, an antagonist of the myostatin receptor (activin-II*β*), is known to prevent the inhibitory effect of myostatin on muscle growth, and follistatin levels are increased by EC supplementation in muscle tissue and serum [35, 36]. Additionally, treatment with EC, ECG, and EGCG significantly influences the expression of MuRF-1; atrogin-1 is upregulated by three-dimensional clinorotation through inhibition of the ERK signaling in mouse C2C12 skeletal myotubes [37]. These results suggest that green tea extract with high a catechins content is a nutritional factor involved in protein synthesis and protein degradation and may influence skeletal muscle mass preservation.

The present results showed age-associated morphological changes were associated with myogenic regulatory factor, muscle oxidative capacity, and skeletal muscle metabolism. In addition, the greatest rates and extent of muscle atrophy were known in the soleus and biceps femoris. Taken together, as compared with epicatechin, tannase-converted green tea extract contributed to a greater improvement in myotube development and protective properties in the soleus and biceps femoris. The effects of *T*_2_ treatment was demonstrated by an improvement in the regulation of muscle regulatory factors, mTOR/S6K pathway, and radical-scavenging ability.

## 5. Conclusion

In conclusion, our results suggest that tannase-converted green tea extract prevents muscle loss and regulates the quantity and quality of muscle by influencing the levels of antioxidant stress-related enzymes and muscle differentiation factors to a greater extent than administration of epicatechin and middle-dose green tea extract. The properties of tannase-converted green tea extract are considered to be a result of its radical-scavenging ability and regulation of skeletal muscle mass-related gene expression and protein levels. Thus, our results suggest that tannase-converted green tea extract with high EC, EGC, and GA contents can be used as a supplement for alleviating muscle loss in aged mice.

## Data Availability

The data used to support the findings of this study are available from the corresponding author upon request.
